# Patients with left ventricular ejection fraction greater than 58 % have fewer incidences of future acute decompensated heart failure admission and all-cause mortality

**DOI:** 10.1007/s00380-015-0657-1

**Published:** 2015-03-14

**Authors:** Toshihiko Goto, Kazuaki Wakami, Hidekatsu Fukuta, Hiroshi Fujita, Tomomitsu Tani, Nobuyuki Ohte

**Affiliations:** Department of Cardio-Renal Medicine and Hypertension, Nagoya City University Graduate School of Medical Sciences, 1 Kawasumi, Mizuho-cho, Mizuho-ku, Nagoya, 467-8601 Japan

**Keywords:** Aortic blood flow, Diastolic dysfunction, Ejection fraction, Inertia stress, Heart failure

## Abstract

Based on our previous observation, inertia stress (IS) of late systolic aortic flow was often observed in left ventricles with relatively higher left ventricular (LV) ejection fraction (EF). Most left ventricles with relatively lower LVEF did not have IS. Accordingly, lack of IS may correlate with LV diastolic dysfunction through the loss of LV elastic recoil and may contribute to the pathogenesis of heart failure (HF) and reduced survival. We enrolled 144 consecutive patients that underwent cardiac catheterization for the diagnosis of coronary artery disease. Left ventricular ejection fraction (LVEF) was obtained from left ventriculography. The IS was calculated from the LV pressure (*P*)−d*P*/d*t* relation. The study endpoint of this retrospective outcome-observational study was combined subsequent acute decompensated heart failure (ADHF) and all-cause mortality. During the follow-up period (median 6.1 years), seven unscheduled hospitalizations for ADHF and nine all-cause deaths were observed. The event-free survival rate was significantly higher among patients with IS than among patients without IS (log-rank, *p* = 0.001). On a multivariate Cox regression analysis, lack of IS was a prime predictor of the endpoint during follow-up (hazard ratio: 6.98; 95 % confidence interval: 1.48–33.03; *p* = 0.01). An LVEF ≥ 58 % was a surrogate indicator for the presence of IS, and patients with LVEF ≥ 58 % had fewer incidences of the endpoint than patients with LVEF < 58 %. In conclusion, lack of IS or LVEF < 58 % should be a predictor of future ADHF and all-cause mortality.

**Electronic supplementary material:**

The online version of this article (doi:10.1007/s00380-015-0657-1) contains supplementary material, which is available to authorized users.

## Introduction

The blood in the aorta, once set in motion, will continue in motion because of inertia until the heart stops it [1]. In late systole, when left ventricular (LV) muscle shortening has reached a limit but its tension-generating ability for the LV wall is still maintained, the inertia of the blood flowing out of the left ventricle causes rapid end-systolic unloading of the left ventricle, producing a much smaller LV end-systolic volume, much greater LV elastic recoil force, and much speeded LV relaxation [2–4]. Inertia stress (IS) is defined as pressure (*P*) decay augmented by the effect of the inertia of blood flowing out of the left ventricle on a phase plot of *P* (d*P*/d*t* vs. *P*) during late systole and early diastole. Thus, lack of IS of late systolic aortic flow is associated with abnormal LV relaxation in patients with coronary artery disease (CAD) and atypical chest pain [4]. LV diastolic dysfunction carries a substantial risk of subsequent development of heart failure (HF) and reduced survival, even when it is asymptomatic [5–11]. IS is observed in left ventricles with good systolic function, but not in all left ventricles with left ventricular ejection fraction (LVEF) ≥ 50 % [4]. LVEF ≥ 50 % has been defined as preserved or normal LV systolic function [9]. Although approximately one-half of patients with HF have preserved LVEF [5, 12], some epidemiological studies have demonstrated a reduced distribution of higher LVEF patients with acute decompensated heart failure (ADHF) [13, 14]. On the other hand, LVEF has been accepted as a prognostic indicator in patients with CAD [15]. Accordingly, this study had two aims: to identify the association of a lack of IS with subsequent ADHF and all-cause mortality in patients that underwent cardiac catheterization for the assessment of CAD; and to investigate whether we were able to predict the existence of IS using LVEF as a surrogate in such patients.

## Materials and methods

### Patients

The study patients consisted of 144 consecutive patients that underwent diagnostic cardiac catheterization to evaluate CAD using a catheter-tipped micromanometer from January 2004 to June 2006. All patients had symptoms suggestive of angina pectoris and/or clinical signs of CAD, including positive electrocardiographic changes during exercise, abnormal myocardial perfusion scintigraphic findings, and a previous history of myocardial infarction (MI) or coronary revascularization. Patients with renal insufficiency (serum creatinine ≥ 1.5 mg/dL), atrial fibrillation or flutter, artificial pacemaker, hemodynamically significant valvular disease, a post-prosthetic valve replacement condition, idiopathic dilated or hypertrophic cardiomyopathy, ADHF, or acute coronary syndrome were excluded. According to the findings of coronary angiography and left ventriculography, 126 patients had CAD: 36 without prior MI and 90 with prior MI. CAD was defined as at least 50 % narrowing of the luminal diameter of one or more of the major coronary arteries, as determined by selective coronary angiography. Prior MI was diagnosed on the finding of localized LV wall motion abnormality using biplane contrast left ventriculography with related electrocardiographic changes. Of the 90 MI patients, 46 had anterior-wall MI, 26 had inferior-wall MI, and 18 had combined anterior- and inferior-wall MI. The remaining 18 patients (those not identified as having CAD) had neither significant coronary stenosis nor LV wall motion abnormality, but had atypical chest pain.

All studies were performed while patients were receiving cardiac medications. In describing patient characteristics, hypertension was defined as systolic blood pressure of at least 140 mmHg and/or diastolic blood pressure of at least 90 mmHg or being treated with antihypertensive drugs. Diabetes mellitus was diagnosed when the fasting blood glucose level was greater than 126 mg/dL or when the patient was treated with blood glucose-lowering medicine. Hypercholesterolemia was defined as a low-density lipoprotein cholesterol level exceeding 140 mg/dL or being treated with cholesterol-lowering medicine. This study was performed in a retrospective manner, and data were collected from our database. The Ethical Guidelines Committee of Nagoya City University Graduate School of Medical Sciences approved the study protocol. Written informed consent for future data analysis regarding the cardiac catheterization data was obtained from all patients at the time of cardiac catheterization.

### Cardiac catheterization

Before contrast material was injected into the left ventricle or coronary arteries, LV pressure (*P*) waves were obtained with a catheter-tipped micromanometer (SPC-454D, Millar Instrument Co., Houston, TX) and recorded on a polygraph system (RMC-3000; Nihon Kohden Inc., Tokyo, Japan) as analog *P* signals. The *P* signals were sent to a digital data recorder (NR-2000, Keyence, Osaka, Japan) at a sampling interval of 2 ms, as we have reported elsewhere [3, 4, 8, 16, 17]. d*P*/d*t* was obtained by differentiating digitally the LV *P* with respect to time, and peak (−d*P*/d*t*) was defined as the maximum negative value of d*P*/d*t*. The time constant Tw was computed by applying a monoexponential fitting with zero asymptote to the LV *P* decay [18]. Another time constant of isovolumic LV *P* decay, Tp, was defined as the negative inverse slope of the relation between d*P/*d*t* and *P* on a phase loop. The intermediate data points between peak (−d*P*/d*t*) and the minimum pressure in the phase plane were fitted to a rectilinear line using the method of least squares, d*P*/d*t*_linear = −*kP* + *C* (*k* > 0),

where *k* and *C* were constants to be estimated.

**Fig. 1:**
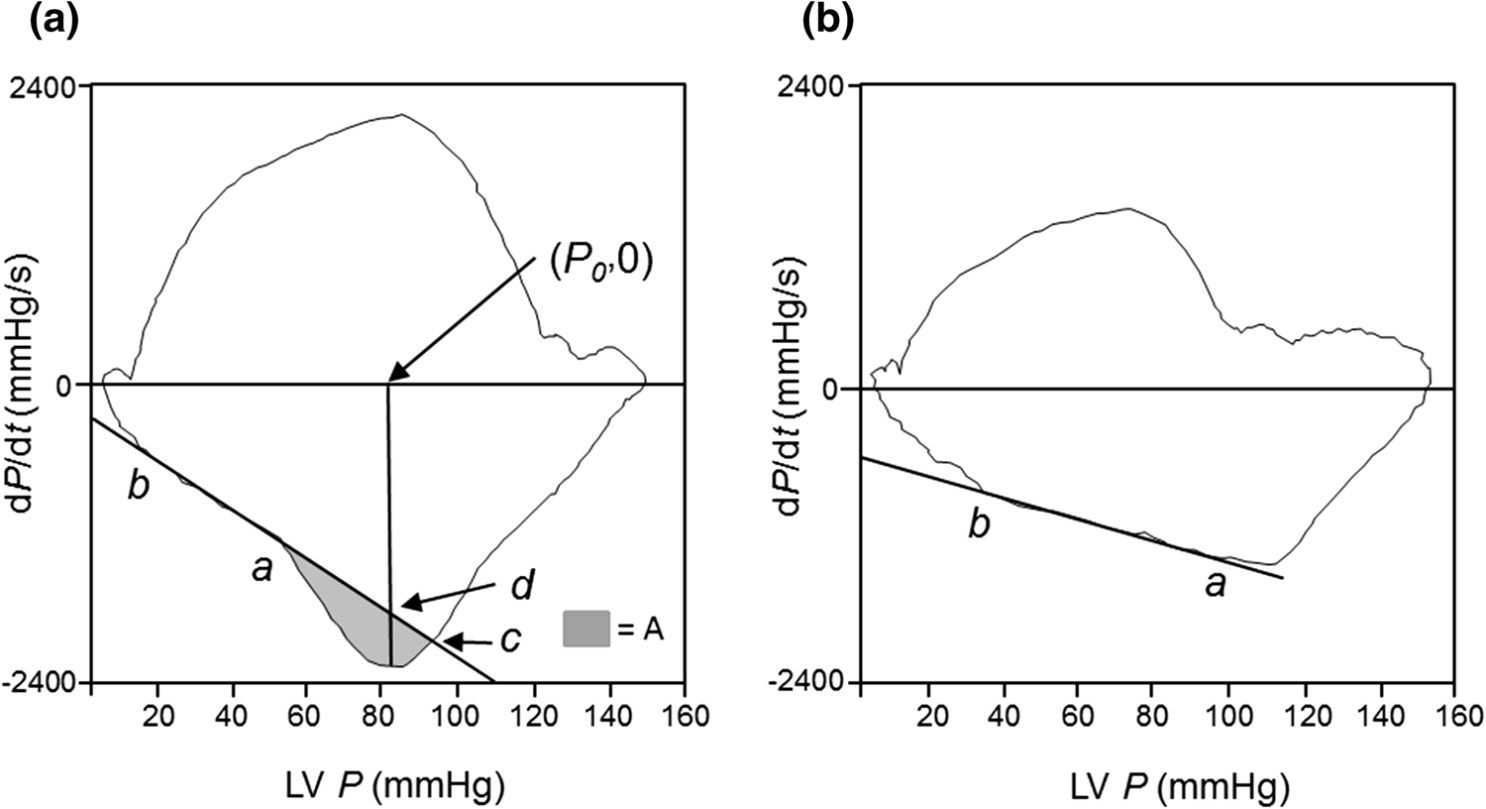
Left ventricular (LV) pressure (*P*)–first derivative of LV *P* (d*P*/d*t*) relationship (phase loop). Loops were obtained from patients with (**a**) and without (**b**) inertia stress (IS). The negative inverse slope of the best linear-fitting line (d*P*/d*t*_linear) between points ***a*** and ***b*** is equal to the time constant of exponential pressure decay during isovolumic relaxation (Tp). The Tp was 56.3 ms in panel **a** and 83.1 ms in panel **b**. *Grey shading area* (*A*) in the *left panel* denotes an area enclosed by the measured *P*−d*P*/d*t* curve and the d*P*/d*t*_linear, A = integral_*P*(a)^∧^ *P*(c)(d*P*/d*t* − d*P*/d*t*_linear)d*P,* where *P*(a) is the pressure at point a and *P*(c) is the pressure at point c which is defined as the intersection of *P*−d*P*/dt curve and dP/dt_linear. The area A divided by the vertical distance between (***P***_0_ **, 0**), and point ***d*** is equal to the amount of pressure decay augmented by the effect of the inertia of blood flowing out of the left ventricle, and is defined as the IS. The IS was 3.6 mmHg (479.9 Pa) in panel **a** and 0.12 mmHg (16.0 Pa) in panel **b**

We excluded the first several data points after peak (−d*P*/d*t*) and those before the minimum *P*, because these data points deviated considerably from the linear fitting. We conducted this exclusion as follows. We took approximately 20 data points on the intercorporate *P*−d*P*/d*t* data starting from peak (−d*P*/d*t*). d*P*/d*t* was obtained at regular sampling intervals of *P*. From these points, we chose approximately 10 successive points, fitted a straight line to this set of points using the method of least squares, and calculated the standard error of the estimate. We repeated this procedure for all possible sets of the same number of successive points, and selected the set that yielded the minimum standard error of the estimate. From this set, we calculated the least squares values of *k* and *C*. The time constant Tp was given by Tp = 1/*k* [2, 4]. The element 1/*k* was computed as the negative inverse slope of the best linear-fitting line (d*P*/d*t*_linear) between points ***a*** and ***b*** (Fig. 1). Points ***a*** and ***b*** indicate the start and end points of the d*P*/d*t*_linear for the slope of the relation between d*P*/d*t* and *P* during isovolumic relaxation. A loop obtained from a patient with IS is shown in Fig. 1a, and a loop obtained from a patient without IS is shown in Fig. 1b. The shaded area A shown in the Fig. 1a is defined as the area between the measured *P*–d*P*/d*t* curve and the d*P*/d*t*_linear,

A = integral_*P*(a)^∧^ *P*(c)(d*P*/d*t* − d*P*/d*t*_linear)d*P*

where *P*(a) is the pressure at point a and *P*(c) is the pressure at point c which is defined as the intersection of *P*–d*P*/dt curve and dP/dt_linear.

The point ***d*** was determined as the crossing point of the vertical line that passes the points (***P***_0_ **, 0**) and peak (−d*P*/d*t*), and the extended d*P*/d*t*_linear that passes the points ***a*** and ***b***. The area divided by the vertical distance between (***P***_0_ **, 0)** and point ***d*** is equal to the amount of pressure decay augmented by the effect of the inertia of blood flowing out of the left ventricle, and is defined as the IS of late systolic aortic flow [2, 4].

IS (mmHg) = A/distance between (***P***_0_ **, 0)** and point ***d***

The theoretical background to calculating IS in the phase plane was proposed by Sugawara et al. [2]. A little fluctuation was observed on the obtained phase loops, so that a small amount of IS may be erroneously calculated in some patients without having an apparently observed bump (grey area, Fig. 1a) in those loops around the time of peak (−d*P*/d*t*). The consensus we reached by reviewing the phase loops in our previous study was that we were able to confirm the existence of the bump in phase loops from patients with computed IS ≥ 0.5 mmHg (66.65 Pa) [4]. Thus, in the present study, we followed the previous definition that patients with computed IS ≥ 0.5 mmHg had IS and patients with calculated IS < 0.5 mmHg did not have IS. LV end-diastolic pressure was also determined. Biplane contrast left ventriculography was performed immediately after the pressure measurements. The method of Chapman et al. [19] was employed to calculate LV end-systolic and end-diastolic volumes. Then, LVEF was determined.

Pressure measurements using a catheter-tipped micromanometer in left-sided cardiac catheterization are essential in order to measure IS. Obtaining these pressure measurements is difficult in daily clinical practice, because the procedure is invasive and using a catheter-tipped micromanometer is considerably expensive. As reported, IS is observed in left ventricles with relatively good systolic function [2, 4]. LVEF is a standardized parameter of LV systolic function that is obtained using echocardiography noninvasively, as well as conventional left ventriculography. Thus, we attempted to seek the best threshold value of LVEF to distinguish whether the left ventricle has IS of late systolic aortic flow using the receiver operating characteristic (ROC) curve analysis.

### Statistical analysis

SPSS statistical software (version 17.0, SPSS Inc., Chicago, IL) was used for statistical analysis. Triglyceride level was summarized as median and interquartile range (IQR) (25th to 75th percentiles). Other data are presented as mean ± standard deviation or frequency (%). Relationships between two parameters were evaluated using Spearman’s correlation coefficients by ranks. Differences in parameters between two groups were compared using Student’s unpaired *t* test or the Mann–Whitney *U* test, as appropriate. A difference in incidence was compared using the Chi-squared test. For event-free survival analysis, Kaplan–Meier curves were generated and compared by use of the log-rank test. We calculated hazard ratios (HRs) derived from the Cox proportional hazard model to identify predictors of combined subsequent HF and all-cause mortality. The variables entered into the model were sex, age ≥65 years, prior MI, LVEF <50 %, Tw ≥ 48 ms, and with or without IS. Differences with *p* < 0.05 were considered statistically significant.

## Results

A total of 144 patients were enrolled (age 65.8 ± 8.9 years, 116 men and 28 women). Clinical characteristics for all patients and subgroup demographics are shown in Tables 1 and 2. There were no significant differences in age, sex, height, weight, mean blood pressure, heart rate, or subscribed medicine between patients with subsequent ADHF and all-cause death and patients without those events. Compared with patients without events during the follow-up period, patients with events had significantly lower LVEF and significantly longer LV relaxation time constants Tw and Tp. The group with events had significantly higher prevalence of prior MI and absence of IS than the group without events.

**Table 1:** Comparisons of clinical characteristics and hemodynamic variables

Characteristic	All patients	Without events^a^	With events	*p*
Number	144	128	16	
Male/female	116/28	102/26	14/2	0.46
Age (years)	65.8 ± 8.7	65.5 ± 8.8	67.8 ± 7.8	0.33
Height (cm)	162.3 ± 8.1	162.3 ± 8.3	162.2 ± 6.1	0.97
Weight (kg)	63.9 ± 10.4	64.4 ± 10.6	60.2 ± 7.0	0.13
Body surface area (m^2^)	1.71 ± 0.16	1.71 ± 0.17	1.66 ± 0.11	0.24
Body mass index (kg/m^2^)	24.2 ± 3.3	24.4 ± 3.3	22.9 ± 2.6	0.09
Heart rate (beats/min)	66.8 ± 10.7	66.7 ± 10.4	67.9 ± 13.2	0.68
Mean blood pressure (mmHg)	93.9 ± 14.1	94.1 ± 14.2	92.2 ± 13.8	0.61
LVEF ejection fraction (%)	62.4 ± 12.4	63.4 ± 12.0	54.8 ± 13.3	0.009
LV end-diastolic pressure (mmHg)	14.1 ± 5.0	13.8 ± 4.7	17.1 ± 5.9	0.012
Tw (ms)	46.0 ± 9.1	45.3 ± 8.9	51.9 ± 8.6	0.005
Tp (ms)	78.0 ± 27.0	75.9 ± 25.8	94.8 ± 31.1	0.008
Lack of IS (%)	25	20.3	61.5	0.005

Data represent mean ± standard deviation or frequency

*LV* left ventricular, *LVEF* left ventricular ejection fraction, *Tw* left ventricular relaxation time constant calculated by Weiss’s method, *Tp* left ventricular relaxation time constant calculated from phase loop, *IS* inertia stress

^a^The combined endpoint was defined as subsequent acute decompensated heart failure and all-cause mortality

**Table 2:** Comparisons of clinical characteristics, underlying diseases, and medications

Characteristic	All patients	Without events	With events	*p*
Total cholesterol (mg/dL)	187.5 ± 36.4	188.1 ± 36.0	190.6 ± 46.8	0.80
Triglycerides (mg/dL)	125 [IQR, 90–189]	128 [IQR, 91–195]	119.5 [IQR, 80.8–154]	0.26
HDL cholesterol (mg/dL)	45.6 ± 13.1	45.6 ± 12.3	44.3 ± 16.2	0.70
LDL cholesterol (mg/dL)	111.6 ± 32.8	112.0 ± 32.2	121.1 ± 37.8	0.30
Glucose (mg/dL)	117.1 ± 45.8	117.7 ± 48.0	112.1 ± 23.0	0.64
HbA1c (%)	6.5 ± 1.9	6.4 ± 1.9	6.6 ± 1.8	0.65
Serum creatinine (mg/dL)	0.90 ± 0.36	0.90 ± 0.37	0.93 ± 0.22	0.77
Hemoglobin (g/dL)	13.3 ± 1.5	13.5 ± 1.5	12.3 ± 2.6	0.06
Hypertension (%)	43.1	43.0	43.8	0.95
Hypercholesterolemia (%)	68.2	68.4	62.5	0.89
Diabetes mellitus (%)	32.6	32.8	31.3	0.90
Prior MI (%)	62.5	59.4	87.5	0.03
Prior heart failure (%)	13.2	14.1	6.3	0.38
Prior PCI (%)	40.3	41.4	31.3	0.44
Prior CABG (%)	8.3	7.8	12.5	0.52
Diuretics (%)	17.6	19.0	6.3	0.21
Statins (%)	59.0	59.4	56.3	0.76
ACEIs or ARBs (%)	35.2	35.9	25.0	0.36
β-blockers (%)	41.5	41.3	43.8	0.85
CCBs (%)	23.9	23.8	25.0	0.92

Data represent mean ± standard deviation or frequency or median and interquartile range (IQR). Blood samples were obatained at fasting

*HDL* high-density lipoprotein, *LDL* low-density lipoprotein, *Hb* hemoglobin, *MI* myocardial infarction, *PCI* percutaneous coronary intervention, *CABG* coronary artery bypass grafting, *BNP* brain natriuretic peptide, *ACEI* angiotensin-converting enzyme inhibitor, *ARB* angiotensin receptor blocker, *CCB* calcium channel blocker

**Fig. 2:**
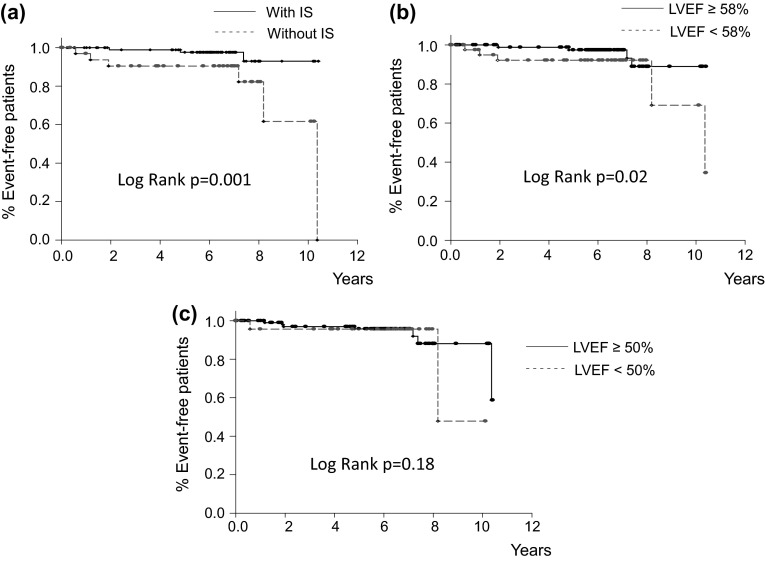
Kaplan–Meier curves for the combined endpoint of subsequent acute decompensated heart failure (ADHF) and all-cause death in patients with inertia stress (IS) (**a**), left ventricular ejection fraction (LVEF) ≥58 % (**b**), and LVEF ≥ 50 % (**c**). The combined endpoint-free survival rate was significantly higher in patients with IS than in those without IS. It was also higher in patients with LVEF ≥58 % than those with LVEF <58 %. However, no significant difference in the endpoint-free survival rate was observed between patients with LVEF ≥50 % and patients with LVEF <50 %

During the follow-up period (median 6.1 years), seven unscheduled hospitalizations due to ADHF and nine all-cause deaths were observed (Table 3). The combined endpoint of subsequent ADHF and all-cause deaths occurred in 6 of 108 patients (5.6 %) with IS and 10 of 36 patients (27.8 %) without IS. The cause of non-cardiac death was cancer in all patients with or without events. Only one case with IS that died due to cardiac death was a sudden death; this patient underwent coronary bypass surgery. A Kaplan–Meier plot showed that the incidence of the combined endpoint of subsequent ADHF and all-cause mortality was significantly lower among patients with IS than among patients without IS (log-rank, *p* = 0.001; Fig. 2a).

**Table 3:** The number of patients that reached the study endpoints

	Patients with IS	Patients without IS	Total patients
Endpoint	108	36	144
All-cause mortality	4	5	9
Cardiac death	1	2	3
Non-cardiac death	3	3	6
Hospitalization due to acute decompensated heart failure	2	5	7

All data are presented as numbers

*IS* inertia stress

**Fig. 3:**
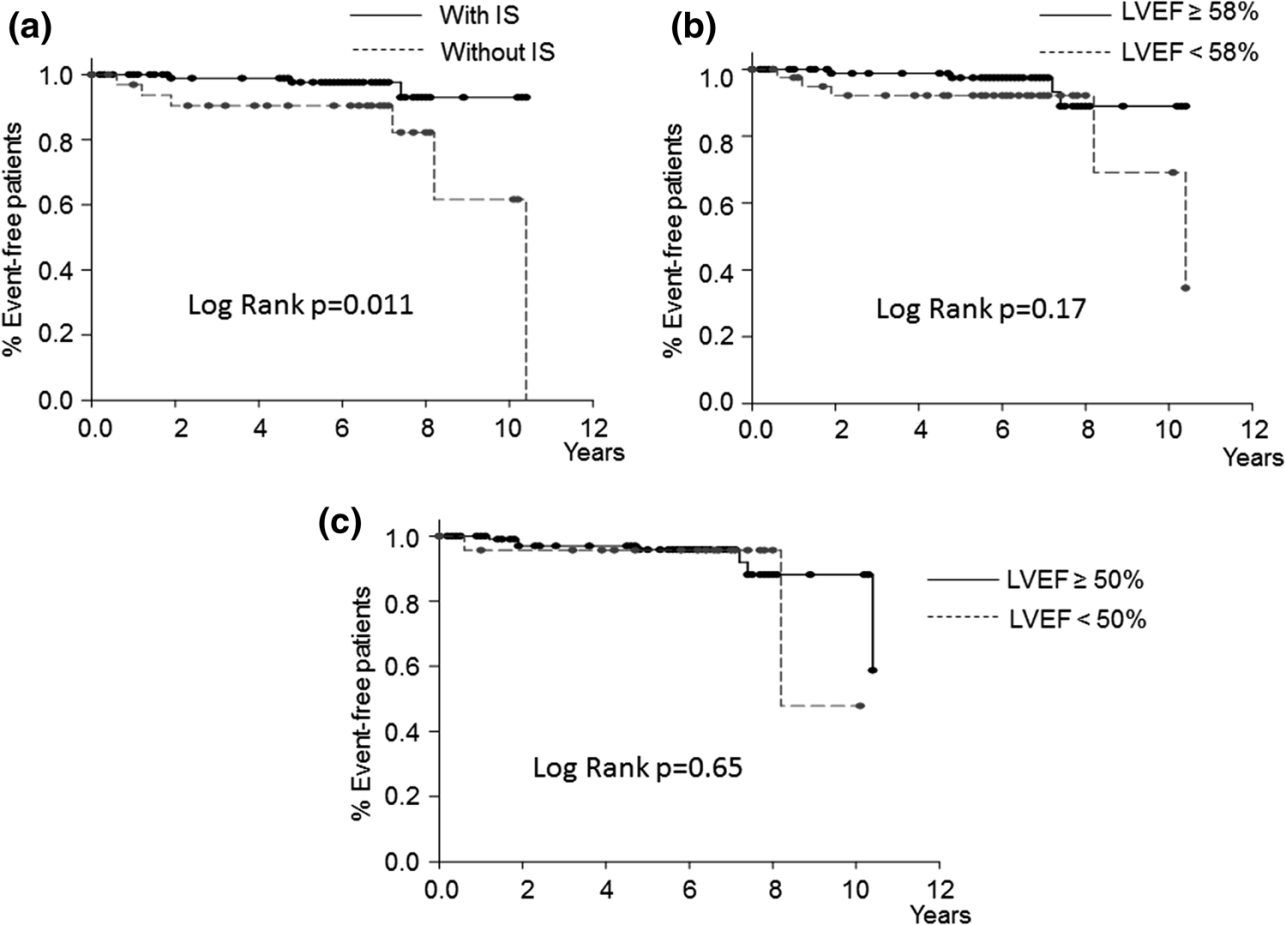
Kaplan–Meier curves for the combined endpoint of subsequent acute decompensated heart failure and cardiac death in patients with inertia stress (IS) (**a**), left ventricular ejection fraction (LVEF) ≥58 % (**b**), and LVEF ≥ 50 % (**c**). The combined endpoint-free survival rate was significantly higher in patients with IS than in patients without IS. No significant differences in the combined endpoint-free survival rate were observed between the patients with LVEF ≥58 % and patients with LVEF <58 %, or between the patients with LVEF ≥50 % and patients with LVEF <50 %

**Fig. 4:**
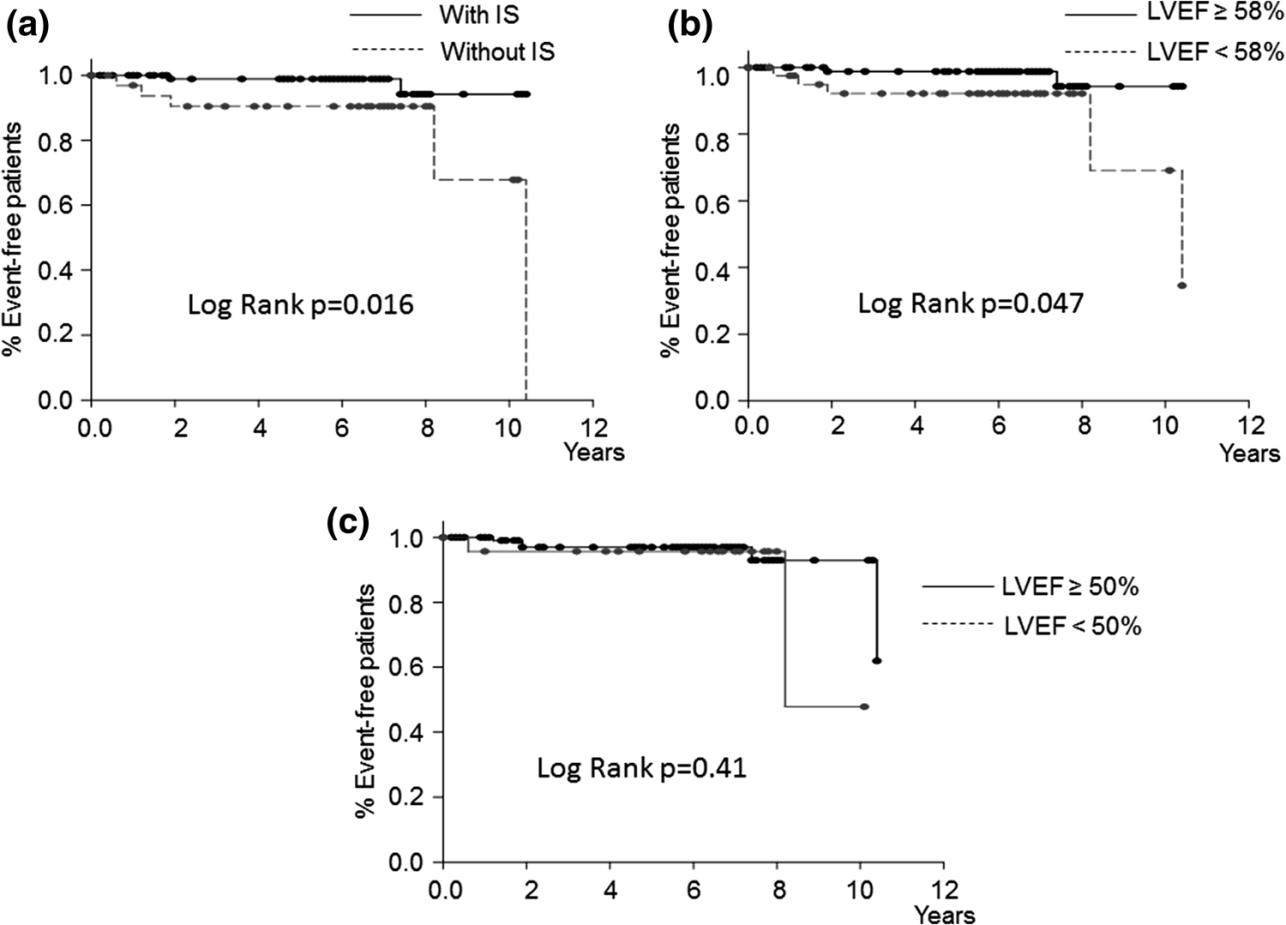
Kaplan–Meier curves for subsequent acute decompensated heart failure (ADHF) in patients with inertia stress (IS) (**a**), left ventricular ejection fraction (LVEF) ≥58 % (**b**), and LVEF ≥50 % (c). ADHF-free survival rate was significantly higher in patients with IS than in patients without IS. It was also higher in patients with LVEF ≥58 % than in patients with LVEF <58 %. No significant difference in ADHF-free survival rate was observed between patients with LVEF ≥50 % and patients with LVEF <50 %

There were also no significant differences in age, sex, height, weight, mean blood pressure, heart rate, or subscribed medicine between patients who reached another combined endpoint of subsequent ADHF and cardiac death and patients who did not (Supplementary Tables S1 and S2). The occurrence of such an endpoint was significantly lower in patients with IS than in patients without IS (log-rank, *p* = 0.011; Fig. 3a). The incidence of subsequent ADHF was lower in patients with IS than in patients without IS (log-rank, *p* = 0.016; Fig. 4a). Again, no significant differences in age, sex, height, weight, mean blood pressure, heart rate, or subscribed medicine were observed between the patient groups (Supplementary Tables S3 and S4). Multivariate Cox regression analyses indicated that lack of IS was the predictor of the combined endpoint of subsequent ADHF and all-cause mortality (HR: 6.98; 95 % confidence interval [CI]: 1.48–33.03; *p* = 0.01), the combined endpoint of subsequent ADHF and cardiac death (HR: 20.3, 95 % CI: 2.37–174, *p* = 0.006), and subsequent ADHF (HR: 22.3, 95 % CI 1.42–350, *p* = 0.03) during the follow-up period (Table 4).

**Table 4:** Factors associated with events in univariate and multivariate analyses

	Univariate			Multivariate		
	Hazard ratio	95 % CI	*p*	Hazard ratio	95 % CI	*p*
Subsequent acute decompensated heart failure and all-cause mortality						
Age ≥ 65 years	1.38	0.48–4.00	0.55			
Male	1.26	0.28–5.58	0.76			
Prior MI	3.24	0.73–14.4	0.12			
LVEF < 50 %	2.18	0.75–6.41	0.16			
Tw ≥ 48 ms	3.40	1.22–9.43	0.02	2.34	0.72–7.58	0.16
Lack of IS	4.65	1.68–12.9	0.003	6.98	1.48–33.0	0.01
Subsequent acute decompensated heart failure and cardiovascular death						
Age ≥ 65 years	2.19	0.45–10.6	0.33			
Male	1.69	0.34–8.38	0.52			
Prior MI	3.39	0.42–27.7	0.25			
LVEF < 50 %	1.44	0.29–7.171	0.65			
Tw ≥ 48 ms	2.64	0.70–10.0	0.15			
Lack of IS	5.15	1.27–20.8	0.02	20.3	2.37–174	0.006
Subsequent acute decompensated heart failure						
Age ≥ 65 years	1.52	0.30–7.88	0.62			
Male	2.50	0.46–13.6	0.29			
Prior MI	2.46	0.29–21.1	0.41			
LVEF < 50 %	2.17	0.40–11.91	0.37			
Tw ≥ 48 ms	2.77	0.63–12.7	0.19			
Lack of IS	6.34	1.22–32.9	0.03	22.3	1.42–350	0.03

*CI* confidence interval, *MI* myocardial infarction, *LVEF* left ventricular ejection fraction, *Tw* left ventricular relaxation time constant calculated by Weiss’s method, *IS* inertia stress

**Fig. 5:**
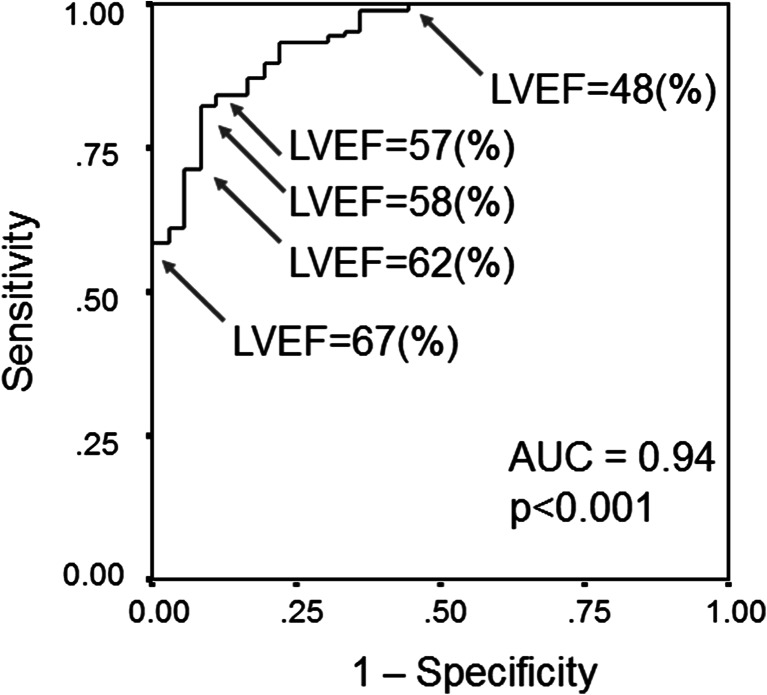
A receiver operating characteristic (ROC) curve for the left ventricular ejection fraction (LVEF) to predict an absence of inertia stress (IS). The area under the curve (AUC) is 0.94 (*p* < 0.001). LVEF values of 57, 58, and 62 % were the candidate LVEF values that were able to predict whether the left ventricle had significant IS with balanced high sensitivity and specificity. An LVEF value of 58 % had both highest sensitivity and highest specificity for this purpose. LVEF <48 % predicted an absence of IS with 100 % sensitivity, while LVEF >67 % predicted an absence of IS with 100 % specificity

The area under the curve (AUC) for LVEF to predict an absence of IS of late systolic aortic flow was 0.94 (95 % CI: 0.90–0.98, *p* < 0.001). From the receiver ROC curve analysis, an LVEF value of 58 % had 87 % sensitivity and 83 % specificity for predicting an absence of IS (Fig. 5). Other LVEF values indicated in the ROC curve were 57 and 62 % (Fig. 5). An LVEF value of 57 % had 87 % sensitivity and 81 % specificity for predicting an absence of IS, and an LVEF value of 62 % had 84 % sensitivity and 83 % specificity for this purpose. Among these three possible threshold values, the LVEF value of 58 % was characterized by both the greatest sensitivity and the greatest specificity. In addition, an LVEF value of 48 % had 100 % sensitivity and 56 % specificity, and an LVEF value of 67 % had 58 % sensitivity and 100 % specificity for predicting an absence of IS. An LVEF value <48 % predicts an absence of IS with negative predictive value of 100 %. An LVEF value >67 % predicts an existence of IS with positive predictive value of 100 %.

Left ventricular ejection fraction <58 % was significantly associated with the combined endpoints of future ADHF and all-cause mortality (log-rank, *p* = 0.02, Fig. 2b); however, it was not significantly associated with the combined endpoint of subsequent ADHF and cardiac death (log-rank, *p* = 0.17, Fig. 3b). LVEF <58 % was also significantly related to the subsequent ADHF (log-rank, *p* = 0.047, Fig. 4b). LVEF <50 % had no significant relationships to these three endpoints (Figs. 2c, 3c, 4c).

## Discussion

In the present study, we demonstrated that lack of IS of late systolic aortic flow is an independent predictor of subsequent ADHF and all-cause mortality in patients with CAD and atypical chest pain. An LVEF value of <58 % was able to detect an absence of IS with adequate sensitivity and specificity. Furthermore, LVEF <58 % was significantly associated with subsequent ADHF and all-cause mortality. However, LVEF <50 % had no significant relationship with this combined endpoint, although LVEF = 50 % is a well-recognized threshold value of LVEF to divide patients into those with preserved LVEF and those with reduced LVEF.

### Effect of IS on prognosis

In this study, patients that were hospitalized for ADHF or with all-cause death had significantly lower LVEF, longer LV relaxation time constants (Tw and Tp), and higher prevalence of lack of IS than patients without these events during the follow-up period. The IS is produced by left ventricles with good systolic function [2]. Thus, lack of IS is related to loss of elastic recoil in the left ventricle, which in turn results in the deterioration of LV relaxation [4]. This means that even with LVEF ≥50 %, the left ventricle would not have good LV systolic function if it does not have IS. In other words, left ventricles with good systolic function enhance their relaxation through the elastic recoil of the left ventricle caused by the IS [4]. Published reports indicate that even in patients with LVEF > 50 %, impaired LV systolic function (which was evaluated using mitral annular excursion during systole or peak mitral annular velocity during systole) is associated with LV diastolic dysfunction and HF [20–22]. In the present study, patients that reached the combined endpoint of subsequent ADHF and all-cause mortality had a higher prevalence of prior MI than those without. This finding is compatible with our previous report that the lack of IS observed in CAD patients frequently accompanies prior MI and LV apical asynergy [4]. Subsequent ADHF was rare among patients with IS, and most patients with IS died because of non-cardiac reasons. Thus, left ventricles with IS are unlikely to develop subsequent ADHF. In a multivariate Cox proportional hazard model, lack of IS was an independent predictor of subsequent ADHF and all-cause mortality. In contrast, LVEF <50 % (a standard threshold of LV systolic dysfunction) and Tw ≥48 ms (a definition of abnormal LV relaxation by the European Society of Cardiology [12]) were not selected as predictors.

LV diastolic dysfunction is reportedly an independent predictor of mortality in patients with acute MI [23] and in patients with HF [5, 24]. LV diastolic dysfunction was also recently demonstrated to be an independent predictor of mortality, even in patients with preserved LVEF [25, 26]. However, in all of those studies, LV diastolic function was noninvasively evaluated using Doppler echocardiography. Only our previous study demonstrated an association between invasively determined LV diastolic function and subsequent HF and cardiac death [8]. In that study, we did not examine the role of IS as a predictor of events; the study endpoints in that study also differed from those in this study.

In addition, LVEF is another powerful predictor of prognosis in patients with MI and in patients with HF [15, 27, 28]. In the present study, the LVEF value of <50 % was not a predictor of the study endpoints. This might be caused by the incorrect setting of the threshold LVEF value to reflect LV systolic dysfunction. In the present study, an LVEF value <48 % predicts an absence of IS with negative predictive value of 100 %; however, its relatively low specificity means that many patients with LVEF >50 % had IS. Previous studies have also demonstrated that LVEF loses its power as a prognostic indicator as the LVEF increases [13, 29].

### LVEF as a surrogate for lack of IS

Because the measurement of IS requires left-sided cardiac catheterization using a catheter-tipped micromanometer, we tried to identify a surrogate for the presence or absence of IS by measuring LVEF, a standard clinical parameter of LV systolic function. The ROC curve analysis indicated that LVEF had significant potential for identifying the presence or absence of IS. The LVEF value of 58 % should be a surrogate for whether or not left ventricles have significant values of IS in patients with CAD and atypical chest pain. Furthermore, patients with LVEF ≥58 % had fewer incidents of subsequent ADHF and good prognosis. In contrast, LVEF ≥50 % did not correlate significantly with the endpoints. Although the low LVEF values are the prognostic indicator, we found that the relatively high LVEF value of 58 % also has prognostic power in our patients. Thus, the presence of IS might be one reason why patients with higher LVEF have fewer incidents of ADHF. In the I-PRESERVE study, which investigated the effect of irbesartan on cardiovascular events in patients with HF with preserved ejection fraction (EF), a significant relationship between LVEF and poor outcome was only observed in patient with LVEF <60 % [29]. The LVEF value of 60 % is similar to our results.

### Clinical implications

Although HF with preserved EF accounts for more than one-half of all HF cases [12–14], effective treatments for patients with HF with preserved EF have not been established [29–32]. Four large trials have studied the effects of drug therapy on HF with preserved EF: the Dig Sub-Study [30], CHARM Preserved [31], PEP-CHF [32], and I-PRESERVE [29]. Those studies focused on patients with LVEF >40 or >45 %. In clinical settings, the LVEF value of 50 % is widely used to categorize patients with HF with preserved EF or reduced EF [12]. In the present study, LVEF ≥50 % had no significant relation to subsequent ADHF or the combined endpoint of subsequent ADHF and all-cause deaths, while most patients with LVEF ≥58 % had IS and good prognosis. Thus, we believe that the meaningful LVEF value to distinguish HF patients with preserved LV systolic function from those with relatively reduced LV systolic function is 58 %. Our proposed threshold LVEF value of 58 % might be a step toward improving the currently difficult situation of treating patients that have HF with preserved EF. The standard drug therapy for HF with reduced EF might work in patients with LVEF <58 %.

### Study limitation

There are several limitations in this study. First, we used contrast enhanced left ventriculography for the measurement of LVEF. This methodology has potential sources of inaccuracy in the measurements of LV volumes, which are caused by irregularities of the surface of LV chamber with trabeculae, papillary muscles, and chordae tendineae. In addition, rapid injection of contrast material and/or specific effects of contrast material during left ventriculography may have affected the LV volume measurements. LV volume quantification using cardiac magnetic resonance imaging is more precise method; however, in the viewpoint of a simultaneous measurement of LV volume and LV pressure, we thought that left ventriculography using a catheter-tipped micromanometer was inevitable way. Second, this was a retrospective outcome-observational study conducted at a single institution with a limited number of patients. Therefore, a prospective study with a large number of patients should be conducted to confirm the present findings; however, we believe that the obtained findings are important for understanding the pathophysiology of patients with HF with preserved EF.

## Conclusion

Lack of IS is a predictor of future ADHF and all-cause mortality. An LVEF value ≥58 % could be used as a surrogate indicator that left ventricles have IS. Patients with LVEF ≥58 % may experience lower incidences of future ADHF and mortality, and they should have good prognosis.

## Electronic supplementary material

Below is the link to the electronic supplementary material.

Supplementary material 1 (PDF 335 kb)
