# Supplementary material for: Patients with left ventricular ejection fraction greater than 58 % have fewer incidences of future acute decompensated heart failure admission and all-cause mortality
Source: Heart Vessels. 2015 Mar 14;31(5):734–43. doi: 10.1007/s00380-015-0657-1 (PMC4850208; doi:10.1007/s00380-015-0657-1)
Supplement: Supplementary file 1 — Supplementary material 1 (PDF 335 kb) [file 380_2015_657_MOESM1_ESM.pdf]

Click here to download Supplementary Document only for Review: SFedit141748_Ohte_STab1-F.doc

Supplementary Table S1 Comparisons of clinical characteristics and hemodynamic variables

Characteristic	All patients	Without events*	With events	P
Number	144	135	9	
Male/female	116/28	109/26	7/2	0.83
Age (years)	65.8 ± 8.7	65.5 ± 8.6	69.2 ± 9.7	0.22
Height (cm)	162.3 ± 8.1	162.3 ± 8.1	161.9 ± 7.8	0.90
Weight (kg)	63.9 ± 10.4	64.0 ± 10.6	62.4 ± 6.8	0.65
Body surface area (m^2)	1.71 ± 0.16	1.71 ± 0.17	1.68 ± 0.12	0.65
Body mass index (kg/m^2)	24.2 ± 3.3	24.3 ± 3.3	23.8 ± 2.3	0.70
Heart rate (beats/min)	66.8 ± 10.7	66.7 ± 10.5	68.8 ± 13.6	0.57
Mean blood pressure (mm Hg)	93.9 ± 14.1	93.7 ± 14.2	96.4 ± 12.8	0.58
LVEF (%)	62.4 ± 12.4	62.7 ± 12.6	57.5 ± 8.1	0.22
LV end-diastolic pressure (mm Hg)	14.1 ± 5.0	13.9 ± 4.7	17.6 ± 7.3	0.03
Tw (ms)	46.0 ± 9.1	45.7 ± 9.0	50.6 ± 8.9	0.12
Tp (ms)	78.0 ± 27.0	76.9 ± 25.7	95.8 ± 39.0	0.04
Lack of IS (%)	25	22.2	66.7	0.003

Data represent mean ± standard deviation or frequency.

∗Events were defined as combined subsequent acute decompensated heart failure and cardiovascular death.

LV left ventricular, *LVEF* left ventricular ejection fraction, *Tw* left ventricular relaxation time constant calculated by Weiss's method, *Tp* left ventricular relaxation time constant calculated from phase loop, *IS* inertia stress

Click here to download Supplementary Document only for Review: SFedit141748_Ohte_STab2-F.doc

Supplementary Table S2 Comparisons of clinical characteristics, underlying diseases, and medications

Characteristic	All patients	Without events*	With events	P
Total cholesterol (mg/dL)	187.5 ± 36.4	186.9 ± 36.1	195.2 ± 41.7	0.51
Triglycerides (mg/dL)	125 [IQR, 90-189]	128 [IQR, 90.5-193]	110 [IQR, 80.8-147]	0.21
HDL cholesterol (mg/dL)	45.6 ± 13.1	45.3 ± 12.4	50.9 ± 21.2	0.24
LDL cholesterol (mg/dL)	111.6 ± 32.8	110.6 ± 32.4	127.1 ± 37.5	0.17
Glucose (mg/dL)	117.1 ± 45.8	117.6 ± 47.0	109.8 ± 19.8	0.62
HbA1c (%)	6.5 ± 1.9	6.4 ± 1.5	6.6 ± 0.7	0.52
Serum creatinine (mg/dL)	0.90 ± 0.36	0.90 ± 0.37	0.93 ± 0.22	0.85
Hemoglobin (g/dL)	13.3 ± 1.5	13.4 ± 1.5	12.3 ± 1.7	0.09
Hypertension (%)	43.1	43.0	44.4	0.93
Hypercholesterolemia (%)	68.2	68.6	62.5	0.72
Diabetes mellitus (%)	32.6	33.3	22.2	0.49
Prior MI (%)	62.5	60.7	88.9	0.09
Prior heart failure (%)	13.2	13.3	11.1	0.85
Prior PCI (%)	40.3	40.7	33.3	0.66
Prior CABG (%)	8.3	8.1	11.1	0.76

Diuretics (%)	17.6	18.0	11.1	0.60
Statins (%)	59.9	60.2	55.6	0.79
ACEIs or ARBs (%)	35.2	36.1	22.2	0.40
β-blockers (%)	41.5	40.6	55.6	0.38
CCBs (%)	23.9	23.3	33.3	0.50

Data represent mean ± standard deviation or frequency or median and interquartile range (IQR).

∗Events were defined as combined subsequent acute decompensated heart failure and cardiovascular death.

HDL high-density lipoprotein, *LDL* low-density lipoprotein, *Hb* hemoglobin, *MI* myocardial infarction, *PCI* percutaneous coronary intervention, *CABG*, coronary artery bypass graft, *BNP* brain natriuretic peptide, *ACEI* angiotensin-converting enzyme inhibitor, *ARB* angiotensin receptor blocker, *CCB* calcium channel blocker

Click here to download Supplementary Document only for Review: SFedit141748_Ohte_STab3-F.doc

Supplementary Table S3 Comparisons of clinical characteristics and hemodynamic variables

Characteristic	All patients	Without events*	With events	P
Number	144	137	7	
Male/female	116/28	111/26	5/2	0.53
Age (years)	65.8 ± 8.7	65.6 ± 8.6	68.3 ± 11.08	0.43
Height (cm)	162.3 ± 8.1	162.2 ± 8.1	163.2 ± 8.5	0.75
Weight (kg)	63.9 ± 10.4	64.0 ± 10.2	63.1 ± 6.5	0.83
Body surface area (m^2)	1.71 ± 0.16	1.71 ± 0.17	1.70 ± 0.13	0.92
Body mass index (kg/m^2)	24.2 ± 3.3	24.3 ± 3.3	23.7 ± 2.3	0.67
Heart rate (beats/min)	66.8 ± 10.7	66.6 ± 10.5	71.0 ± 13.9	0.29
Mean blood pressure (mm Hg)	93.9 ± 14.1	93.8 ± 14.4	94.9 ± 13.9	0.85
LVEF (%)	62.4 ± 12.4	62.7 ± 12.5	55.7 ± 8.2	0.14
LV end-diastolic pressure (mm Hg)	14.1 ± 5.0	14.0 ± 4.8	16.4 ± 7.6	0.21
Tw (ms)	46.0 ± 9.1	45.8 ± 9.0	50.8 ± 9.9	0.15
Tp (ms)	78.0 ± 27.0	76.6 ± 25.6	105.4 ± 39.4	0.006
Lack of IS (%)	25	22.6	71.4	0.004

Data represent mean ± standard deviation or frequency.

*Events were defined as subsequent acute decompensated heart failure.

LV left ventricular, *LVEF* left ventricular ejection fraction, *Tw* left ventricular relaxation time constant calculated by Weiss's method, *Tp* left ventricular relaxation time constant calculated from phase loop, *IS* inertia stress

Click here to download Supplementary Document only for Review: SFedit141748_Ohte_STab4-F.doc

Supplementary Table S4 Comparisons of clinical characteristics, underlying diseases, and medications

Characteristic	All patients	Without events*	With events	P
Total cholesterol (mg/dL)	187.5 ± 36.4	187.0 ± 35.9	195.1 ± 47.1	0.57
Triglycerides (mg/dL)	125 [IQR, 90-189]	126 [IQR, 91.5-191]	83.0 [IQR, 74.0-168]	0.24
HDL cholesterol (mg/dL)	45.6 ± 13.1	45.5 ± 12.1	47.2 ± 13.2	0.77
LDL cholesterol (mg/dL)	111.6 ± 32.8	110.6 ± 32.2	132.8 ± 41.5	0.11
Glucose (mg/dL)	117.1 ± 45.8	117.3 ± 46.7	112.0 ± 21.7	0.77
HbA1c (%)	6.5 ± 1.9	6.4 ± 1.5	6.6 ± 0.7	0.77
Serum creatinine (mg/dL)	0.90 ± 0.36	0.90 ± 0.37	0.84 ± 0.15	0.67
Hemoglobin (g/dL)	13.3 ± 1.5	13.4 ± 1.5	11.9 ± 1.6	0.04
Hypertension (%)	43.1	43.1	42.9	0.99
Hypercholesterolemia (%)	68.2	68.3	66.7	0.93
Diabetes mellitus (%)	32.6	32.8	28.6	0.81
Prior MI (%)	62.5	61.3	85.7	0.19
Prior heart failure (%)	13.2	13.1	14.3	0.93
Prior PCI (%)	40.3	40.9	28.6	0.52
Prior CABG (%)	8.3	8.0	14.3	0.56

Diuretics (%)	17.6	18.5	0	0.21
Statins (%)	59.9	60.0	57.1	0.88
ACEIs or ARBs (%)	35.2	36.3	14.3	0.24
β-blockers (%)	41.5	41.5	42.9	0.94
CCBs (%)	23.9	23.7	28.6	0.77

Data represent mean ± standard deviation or frequency or median and interquartile range (IQR).

∗Events were defined as subsequent acute decompensated heart failure.

HDL high-density lipoprotein, *LDL* low-density lipoprotein, *Hb* hemoglobin, *MI* myocardial infarction, *PCI* percutaneous coronary intervention, *CABG*, coronary artery bypass graft, *BNP* brain natriuretic peptide, *ACEI* angiotensin-converting enzyme inhibitor, *ARB* angiotensin receptor blocker, *CCB* calcium channel blocker
